# Leisure-time physical activity and risk of sudden cardiac death: a 28-year follow-up from the Copenhagen City Heart Study

**DOI:** 10.1016/j.eclinm.2026.103825

**Published:** 2026-03-10

**Authors:** Shotaro Isozaki, Tobias Skjelbred, Peder Emil Warming, Eleonora Casarini, Eva Irene Bossano Prescott, Reza Jabbari, Jasmin Mujkanovic, Jacob Tfelt-Hansen

**Affiliations:** a Department of Cardiology, The Heart Centre, Copenhagen University Hospital - Rigshospitalet, 2100, Copenhagen, Denmark; b Section of Forensic Genetics, Department of Forensic Medicine, Copenhagen University, 2100, Copenhagen, Denmark; c Department of Forensic Medicine, Tokai University School of Medicine, 259-1193, Isehara, Japan; d Department of Cardiology, Copenhagen University Hospital - Bispebjerg and Frederiksberg, Bispebjerg Bakke 23, 2400, Copenhagen, Denmark

**Keywords:** Sudden cardiac death, Exercise, Proportional hazards models, Population attributable risk

## Abstract

**Background:**

Sudden cardiac death (SCD) remains a major public health challenge, yet the long-term association of leisure-time physical activity with SCD risk is not well established. We examined whether self-reported leisure-time physical activity is associated with SCD incidence over 28 years, accounting for competing risks from non-SCD mortality and time-updated changes in leisure-time physical activity.

**Methods:**

We included 10,100 participants from the Copenhagen City Heart Study, examined in 1991–1994 and followed until 31 December 2021. SCD events were identified from Danish death certificates using a standardized protocol. Leisure-time physical activity was self-reported at baseline and at 10-year follow-up and incorporated as a time-updated exposure, categorized as low, moderate, or high. Cause-specific Cox models estimated associations with SCD, adjusting for age, sex, smoking, alcohol, and socioeconomic factors. Cumulative incidence was standardized to the Danish population, and population attributable risk for low activity was calculated. This study is registered at ClinicalTrials.gov (NCT02993172).

**Findings:**

During a median follow-up of 28.6 years, 897 SCD events occurred among 10,100 participants (mean age 60.8 years; 56% women). Twenty-year standardized cumulative incidence rose with lower activity. In time-updated analyses, moderate and high leisure-time physical activity were associated with lower SCD risk compared with low activity (hazard ratio (HR) of 0.60, 95% confidence interval (CI) 0.50–0.72; and HR 0.50, 95% CI 0.41–0.62, respectively). After 25 years, 33% (95% CI 21–46%) of SCD could be attributed to low activity.

**Interpretation:**

Higher leisure-time physical activity is associated with lower long-term risk of SCD in this observational cohort. These findings support the potential public health relevance of promoting leisure-time physical activity, although causality cannot be established.

**Funding:**

Research Fund of Tokai University Educational System, Asahikawa Medical University Alumni Fund, 10.13039/100007405Danish Heart Foundation, Birthe and John Meyer Family Foundation, 10.13039/501100009708Novo Nordisk Foundation.

**Research in context**

**Evidence before this study**

Sudden cardiac death (SCD) accounts for a substantial proportion of cardiovascular deaths, yet its epidemiology differs from those of overall cardiovascular or all-cause mortality. Prior systematic reviews and meta-analyses have consistently shown that higher levels of leisure-time physical activity are associated with lower risks of all-cause and cardiovascular mortality. However, evidence specifically linking habitual physical activity to long-term risk of SCD remains limited. In addition, few studies have explicitly accounted for competing risks from non-SCD deaths, and time-updated assessments of physical activity have rarely been incorporated. Consequently, uncertainty remains regarding the long-term association between habitual leisure-time physical activity and rigorously adjudicated SCD risk, as well as its potential population-level impact.

**Added value of this study**

Using a large, population-based cohort with long-term follow-up and rigorously adjudicated SCD events, we found that higher levels of leisure-time physical activity were associated with a lower long-term risk of SCD. By accounting for competing risks from non-SCD deaths, we provide population-standardized cumulative incidence estimates that reflect real-world risk over extended follow-up.

Analyses incorporating repeated assessments of leisure-time physical activity showed that associations were slightly stronger when physical activity was treated as a time-updated exposure, suggesting that more recent activity levels may be particularly relevant for SCD risk. In addition, we estimated that a substantial proportion of SCD events in the population could be attributed to low leisure-time physical activity over long-term follow-up. Together, these findings add to prior observational evidence by providing population-representative estimates that account for competing risks and changes in physical activity over time.

**Implications of all the available evidence**

Collectively, available evidence indicates that higher levels of leisure-time physical activity are associated with a lower long-term risk of SCD. Given that low leisure-time physical activity accounted for about one-third of SCD cases in this cohort, public health strategies promoting active lifestyles could meaningfully reduce the burden of SCD. While causal inference cannot be established from observational data, these findings reinforce the role of regular physical activity as a cornerstone of cardiovascular health promotion and primary prevention at the population level.

## Introduction

Sudden cardiac death (SCD) represents a major public health challenge, accounting for approximately 10–20% of all deaths and nearly 50% of all cardiovascular deaths worldwide.^1^ Unlike all-cause or overall cardiovascular mortality, SCD has distinct underlying mechanisms, risk factors, and clinical implications, and therefore warrants independent investigation in epidemiological studies.^2^^,^^3^ The unpredictable nature of SCD, often occurring without prior symptoms or warning signs, makes it difficult to identify individuals at imminent risk.^4^ Consequently, identifying modifiable risk factors for SCD, and understanding the magnitude of their contribution, is critical for developing effective prevention strategies.

Leisure-time physical activity has been proposed as a protective factor against SCD; however, the existing evidence remains limited and methodologically heterogeneous.^5^ We have observed that young people that do either competitive sport or leisure sport have lower incidence of SCD compared to general population.^6^ Whereas others have reported an increased risk of SCD among young competitive athletes engaged in organized, high-intensity sports requiring regular training and competition, particularly in the presence of underlying cardiomyopathies.^7^ These apparently conflicting findings likely reflect differences in study populations, exposure definitions, outcome ascertainment, and the distinction between acute exercise-related risk and long-term effects of habitual physical activity.

Importantly, most prior population-based studies examining physical activity and cardiovascular outcomes have focused on all-cause or overall cardiovascular mortality, with relatively few specifically addressing SCD as a distinct endpoint.^8^ When SCD has been examined, outcome definitions have often relied on registry-based cause-of-death codes without systematic validation, raising concerns about misclassification. In addition, leisure-time physical activity has typically been assessed only at baseline, without accounting for changes over time, which may introduce time-dependent exposure misclassification and bias risk estimates.^9^^,^^10^ Furthermore, few studies have explicitly accounted for competing risks, such as non-SCD cardiovascular or non-cardiovascular deaths, which are particularly relevant in long-term follow-up and may distort cause-specific hazard estimates if ignored.^11^

In this study, our focus is on the long-term impact of an active lifestyle, rather than SCD occurring acutely during vigorous exertion. While intense exercise can transiently increase the risk of arrhythmic events particularly in younger individuals or those with underlying structural heart disease such as cardiomyopathies, coronary anomalies or obstructive coronary artery disease, the absolute risk is very low.^12^^,^^13^ In contrast, habitual physical activity is consistently associated with a reduction in long-term cardiovascular risk.^14^, ^15^, ^16^

To address these gaps, we aimed to investigate the association between self-reported leisure-time physical activity and the risk of SCD in a large, population-based cohort. Our study is distinguished by a rigorous manual adjudication of SCD based on comprehensive review of death certificates,^17^ repeated assessments of leisure-time physical activity and key covariates enabling time-updated analyses, and explicit handling of competing risks through cause-specific Cox regression models. Together, these features allow a more precise and etiologically relevant assessment of the long-term relationship between habitual physical activity and SCD risk.

## Methods

### Study population

We used data from the Copenhagen City Heart Study, a population-based cohort initiated in 1976 in the Østerbro district of Copenhagen, Denmark. Baseline enrollment included randomly selected adult residents, with follow-up examinations performed approximately every 10 years. At each follow-up, additional participants were invited to join the study to maintain the representativeness of the cohort. The methods of sampling, enrolment, response rates, examination procedures, and follow-up have been described in detail elsewhere.^17^^,^^18^ Information on participants’ history of cardiovascular disease was obtained from the Danish National Patient Register; the detailed definitions and data extraction methods have been described previously.^17^ All Danish residents are assigned a unique Civil Personal Registration (CPR) number, enabling linkage across national health registries. For this study, questionnaire data from the third (1991–1994; hereafter “baseline”) and fourth (2001–2003; “follow-up”) examination rounds of the Copenhagen City Heart Study were linked to the Danish Register of Causes of Death and The National Patient Register by linkage of CPR numbers.^19^

In Denmark, when a death occurs, a medical doctor issues the death certificate and determines the immediate, contributory, and underlying causes of death based on all available information, including medical files. If a death is sudden and unexpected, the police become involved to decide whether a medicolegal external examination should be conducted. This examination is performed jointly by the police and a medical officer of public health (a certified physician), who has access to the body of the deceased for external inspection, first responder records, medical files, and the complete police record, including any eyewitness statements. Information from all these sources is summarized in a supplementary field on the death certificate, rendering Danish death certificates highly suitable for identifying sudden and unexpected deaths.

If the cause of death remains unclear, a forensic autopsy is performed at one of the three forensic departments in Denmark. Alternatively, in cases where the police do not request a forensic autopsy, a hospital autopsy can be conducted at the request of a physician or the next of kin. Hospital autopsies are focused on uncovering the cause of death and are not as rigorous as forensic autopsies. This systematic and detailed documentation ensures high-quality ascertainment of SCD in Denmark.

Participants in our study were followed from the date of their baseline examination, until 31 December 2021 or death, whichever came first. Because cause of death information was only available from 1 January 1993 onwards, follow-up was defined as starting on that date for individuals examined in 1991 or 1992.

### Definition of SCD and outcome ascertainment

SCD was classified into definite, probable, and possible cases according to a standardized review protocol adapted in line with recently published definitions.^20^ Two physicians independently adjudicated all deaths using death certificates, autopsy reports if performed, discharge summaries, and national registry data, with an inter-reviewer agreement rate of 92%. In case of disagreement, consensus was reached through joint review. For the primary analyses, all definite, probable, and possible SCD cases were included as SCD events, while non-SCD deaths were treated as competing events. Participants with incomplete or missing death certificates were censored at the date of their last recorded healthcare contact. A detailed description of the SCD classification criteria is provided in the Supplementary Method S1.

### Exposure assessment

Leisure-time physical activity was assessed using self-administered questionnaires at both baseline (round 3) and follow-up (round 4) examinations. Participants were asked about their physical activity during leisure time, including transport to and from work, over the preceding year. Leisure-time physical activity was categorized into three levels: Low, Moderate, and High. Definitions of each category and the exact questionnaire items used to classify activity are included in Supplementary Methods S2. For baseline analyses, we used round 3 data as this examination was the first to include detailed assessments of leisure-time physical activity along with comprehensive information on socioeconomic, lifestyle, and clinical factors required for multivariable adjustment. For time-dependent analyses, physical activity status from round 4, collected approximately 10 years after baseline, was used to update exposure status. Participants who did not complete the follow-up questionnaire were assumed to have maintained their baseline activity level.

Information on covariates, including alcohol consumption, smoking status, income, and education, was collected at the baseline. Alcohol consumption (beer, wine, and spirits) was converted to total weekly intake in units and categorized into four groups: *none* (0 units per week); Low (1–10 units per week); High (11–20 units per week); and Very High (>20 units per week). Other covariates were categorized according to definitions used in previous publications.^21^ This observational study followed the STROBE reporting guideline.

### Informed consent and ethical approval

Participation in the Copenhagen City Heart Study was based on informed consent. The study was approved by the relevant ethics committees (H-KF-01-144/01) and is registered at ClinicalTrials.gov (NCT02993172). Follow-up data, including hospital discharge summaries and death certificates used for SCD adjudication, were obtained with approval from the Danish Health Data Authority (FSEID-00003293) and the Danish Patient Safety Authority (3-3013-2730/1). The study was further approved by the data-responsible institute (the Capital Region of Denmark, P-2019-159) in accordance with the General Data Protection Regulation.

### Statistical analysis

Median follow-up was calculated using the reverse Kaplan–Meier method.^22^ We predicted the sex-stratified cumulative incidence across categories of leisure-time physical activity by Cause specific Cox (CSC) models while standardizing to the age distribution of the Danish population in 1993 (https://www.dst.dk). In these analyses, deaths from causes other than sudden cardiac death were treated as competing events and individuals were censored at the time of non-SCD death. Missing data were handled using multiple imputation by chained equations, generating 20 imputed datasets. For each imputed dataset, uncertainty in the cumulative incidence estimates was quantified using 1000 nonparametric bootstrap resamples, yielding within-imputation variances. Point estimates and variances from each dataset were then combined using Rubin's rules^23^, ^24^, ^25^ to obtain pooled cumulative incidence estimates and 95% confidence intervals that account for both imputation and bootstrap uncertainty.

Covariate adjustment was guided by a directed acyclic graph (DAG) that we constructed to represent the hypothesized relationships among leisure-time physical activity, potential confounders, mediators, and the risk of sudden cardiac death. The DAG illustrating these hypothesized relationships is provided in Supplementary Figure S1. The DAG was assumed temporal ordering between variables, rather than by statistical testing. The DAG indicated that socioeconomic factors, smoking, alcohol consumption, age, and sex act as confounders, while cardiovascular disease, body mass index (BMI) and diabetes use lie on the causal pathway as potential mediators. Based on this framework, we specified two models. Model 1, which served as the main model, was adjusted for age (continuous, modeled linearly), sex, socioeconomic variables (income and education), alcohol consumption, and smoking status, thereby estimating the total effect of leisure-time physical activity on SCD. Model 2, used as a secondary analysis, additionally adjusted for cardiovascular disease, BMI and diabetes allowing exploration of the extent to which cardiometabolic factors may mediate the association. To explore the extent to which cardiometabolic factors may mediate the association between leisure-time physical activity and SCD, we applied the Difference method.^26^, ^27^, ^28^ Specifically, the log hazard ratio difference between Model 1 and Model 2 was calculated to estimate the proportion of the association potentially explained by these mediators. This approach enabled us to distinguish between the overall impact of physical activity on SCD (Model 1) and the effect that is not explained by established cardiometabolic mediators (Model 2).

We also conducted a dose–response analysis using a CSC model. Leisure-time physical activity was quantified as total weekly energy expenditure in metabolic equivalent task minutes per week (MET-min/week), calculated from questionnaire-based self-reported leisure-time physical activity by assigning standardized MET values to each activity using the 2024 Adult Compendium of Physical Activities and combining reported duration and frequency (Supplementary Table S2). Non-linearity in the association between leisure-time physical activity and SCD was modeled using restricted cubic splines with three knots placed at default quantiles of the leisure-time physical activity distribution, adjusted for Model 1 covariates. Predicted values were exponentiated to obtain hazard ratios (HRs) and normalized to the hazard at leisure-time physical activity = 0 MET-min/week, which served as the reference. The dose–response relationship was visualized by plotting the adjusted HR curve along with its 95% confidence interval (CI).

Time-dependent CSC analyses were conducted using a time-splitting approach to incorporate changes in leisure-time physical activity status between baseline and follow up surveys. For participants with follow up data, the first interval extended from baseline to the follow up visit, and the second interval extended from the follow up visit to the event or censoring. Leisure-time physical activity status, as well as BMI, diabetes, smoking status, and alcohol consumption, were updated at the start of the second interval if follow-up data were available; otherwise, baseline values were carried forward. Sex, educational attainment, history of cardiovascular disorders and income were treated as fixed at baseline. Age was used as the underlying time scale to appropriately account for the effect of aging on sudden cardiac death risk. In addition to the primary time-dependent analysis using repeated measurements of leisure-time physical activity, we also evaluated changes in leisure-time physical activity between two examination cycles. Participants were categorized as having decreased, sustained, or increased leisure-time physical activity. Cause specific cox proportional hazards models were then used to estimate hazard ratios for SCD according to leisure-time physical activity change category. The proportional hazards assumption was evaluated using Schoenfeld residuals.

To evaluate the robustness of our findings, we conducted four sensitivity analyses. Specifically, we restricted the outcome definition to definite and probable SCD cases, performed a complete-case analysis by excluding participants with missing covariates, excluded SCD events occurring within the first year of follow-up in order to minimize the potential for reverse causality and repeated the time-updated analyses after restricting the sample to participants who attended the follow-up examination.

The population attributable risk (PAR) for SCD associated with low leisure-time physical activity was estimated from cumulative incidence functions derived from CSC models. The observed distribution of leisure-time physical activity in the study population was used as the reference.

Standardization was performed using the age and sex distribution of the Danish population in 1993. To quantify the uncertainty of PAR estimates, bootstrap resampling with 1000 iterations was used for variance estimation within each imputed dataset. PAR was calculated under the hypothetical scenario in which all individuals were assigned to the high physical activity category, using the formula:

$$PAR=1\;-\;\frac{R_{all\;high}}{R_{current}}$$

where $R_{current}$ represents the average cumulative incidence in the population based on observed activity proportions, and $R_{all\;high}$ represents the average cumulative incidence if all participants had high physical activity. Estimates across the multiple imputed datasets were combined using Rubin's rules to account for imputation uncertainty.

To explore possible physiological mediators linking leisure-time physical activity with the risk of SCD, we examined six cardiometabolic indicators (Systolic blood pressure (SBP), Resting heart rate (RHR), BMI, total cholesterol (TCHO), High Density Lipoprotein (HDL), and creatinine (Cre)) as outcomes. Linear regression models were fitted for each outcome, adjusting for age, sex, education level, income category, smoking status, and alcohol consumption. Estimated marginal means with 95% confidence intervals for each physical activity group were calculated.

All analyses were conducted using R version 4.5.0 (R Foundation for Statistical Computing).^29^ Two-sided p-values <0.05 were considered statistically significant.

### Role of the funding source

The funder of the study had no role in study design, data collection, data analysis, data interpretation, or writing of the report.

## Results

### Baseline characteristics

By the time of the baseline examination (1991–1994), 10,135 individuals attended, of whom 10,100 were alive on 1 January 1993 and therefore eligible for follow-up. The median follow-up time was 28.6 years (interquartile range, 28.1–29.0 years). The proportion of missing covariate data was less than 5%. For the main exposure, leisure-time physical activity, data were missing for 145 participants (1.4%) (Supplementary Table S1).

**Fig. 1:**
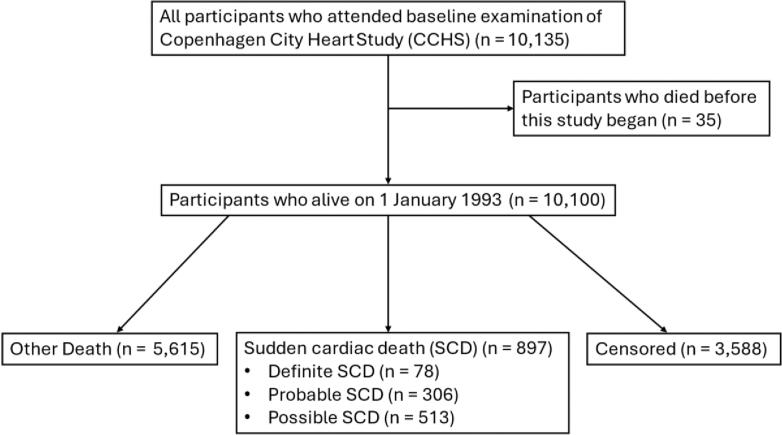
**Flowchart of study participants**. Flow diagram illustrating the selection of study participants from the Copenhagen City Heart Study.

During follow-up, 6512 participants died from any cause, and 142 were censored at the date of last known contact due to incomplete or missing death certificates. We identified 897 SCD events, comprising 78 definite, 306 probable, and 513 possible cases (Fig. 1).

Baseline characteristics by leisure-time physical activity level are shown in Table 1. Overall, the study population comprised 5589 women (56%) and 4366 men (44%), with a mean age of 60.8 years at baseline. In both women and men, higher levels of leisure-time physical activity were associated with younger age, higher educational attainment, and higher income. Among both men and women, higher levels of leisure-time physical activity were associated with a more favorable cardiovascular risk profile, including lower body mass index, lower prevalence of hypertension and diabetes, and a lower prevalence of prior cardiovascular disease. Notably, the prevalence of prior myocardial infarction decreased significantly across physical activity categories in women (p < 0.001), whereas no statistically significant difference was observed among men (p = 0.052). Overall, apart from the sex-specific statistical significance observed for prior myocardial infarction, associations between leisure-time physical activity level and baseline characteristics, including cardiovascular disease (CVD) history, were largely consistent in direction for women and men. Alcohol consumption patterns also varied by physical activity level in a similar manner in women and men, with lower proportions of abstainers and higher proportions of moderate to high alcohol intake observed in the more physically active groups.

**Table 1:** Clinical and demographic characteristics at baseline.

Leisure-time physical activity	Female				Male			
	Low N = 749^a^	Moderate N = 3263^a^	High N = 1577^a^	p-value^b^	Low N = 588^a^	Moderate N = 2021^a^	High N = 1757^a^	p-value^b^
**Age**	68.5 (56.1–76.3)	63.3 (51.5–73.0)	57.7 (43.2–68.6)	<0.001	62.7 (50.4–73.0)	59.9 (49.2–71.0)	56.9 (41.4–68.9)	<0.001
**Smoking**				0.009				<0.001
Never	204 (27%)	1025 (31%)	503 (32%)		69 (12%)	327 (16%)	381 (22%)	
Former	162 (22%)	751 (23%)	392 (25%)		160 (27%)	601 (30%)	525 (30%)	
Current	382 (51%)	1487 (46%)	680 (43%)		359 (61%)	1093 (54%)	851 (48%)	
**Alcohol**				<0.001				<0.001
None	330 (44%)	1012 (31%)	352 (22%)		116 (20%)	239 (12%)	198 (11%)	
Low	310 (41%)	1727 (53%)	895 (57%)		221 (38%)	827 (41%)	736 (42%)	
High	64 (8.6%)	361 (11%)	242 (15%)		106 (18%)	458 (23%)	431 (25%)	
Very high	43 (5.8%)	150 (4.6%)	83 (5.3%)		139 (24%)	480 (24%)	370 (21%)	
**Education**				<0.001				<0.001
No education	363 (51%)	1005 (31%)	343 (22%)		185 (33%)	361 (18%)	253 (15%)	
Apprentice	106 (15%)	548 (17%)	235 (15%)		202 (36%)	823 (41%)	681 (40%)	
Short education	170 (24%)	1034 (32%)	525 (34%)		82 (14%)	315 (16%)	285 (17%)	
Long education	77 (11%)	608 (19%)	428 (28%)		98 (17%)	493 (25%)	475 (28%)	
**Income (DKK/year)**				<0.001				<0.001
0–100,000	419 (59%)	1258 (40%)	476 (31%)		260 (45%)	661 (33%)	512 (29%)	
100,000–200,000	182 (25%)	1204 (38%)	592 (39%)		182 (32%)	765 (39%)	696 (40%)	
200,000-	113 (16%)	700 (22%)	469 (31%)		135 (23%)	556 (28%)	528 (30%)	
**Cardiovascular disease**	425 (57%)	1582 (48%)	608 (39%)	<0.001	361 (61%)	1081 (53%)	875 (50%)	<0.001
**Prior myocardial infarction**	35 (4.7%)	84 (2.6%)	18 (1.1%)	<0.001	44 (7.5%)	120 (5.9%)	86 (4.9%)	0.052
**Antihypertensive medication**	122 (17%)	451 (14%)	136 (8.7%)	<0.001	72 (12%)	256 (13%)	135 (7.7%)	<0.001
**Diabetes**	32 (4.3%)	77 (2.4%)	31 (2.0%)	0.003	47 (8.0%)	101 (5.0%)	58 (3.3%)	<0.001
**Body mass index (kg/m^2^)**	25.6 (22.8–29.8)	24.6 (22.0–28.0)	23.6 (21.6–26.6)	<0.001	26.3 (23.6–29.4)	26.0 (23.6–28.6)	25.2 (23.1–27.7)	<0.001

145 cases omitted due to lack of Physical activity information.

a Median (Q1-Q3); n (%).

b Kruskal–Wallis rank sum test; Pearson's Chi-squared test.

### Association between leisure-time physical activity and risk of SCD

**Fig. 2:**
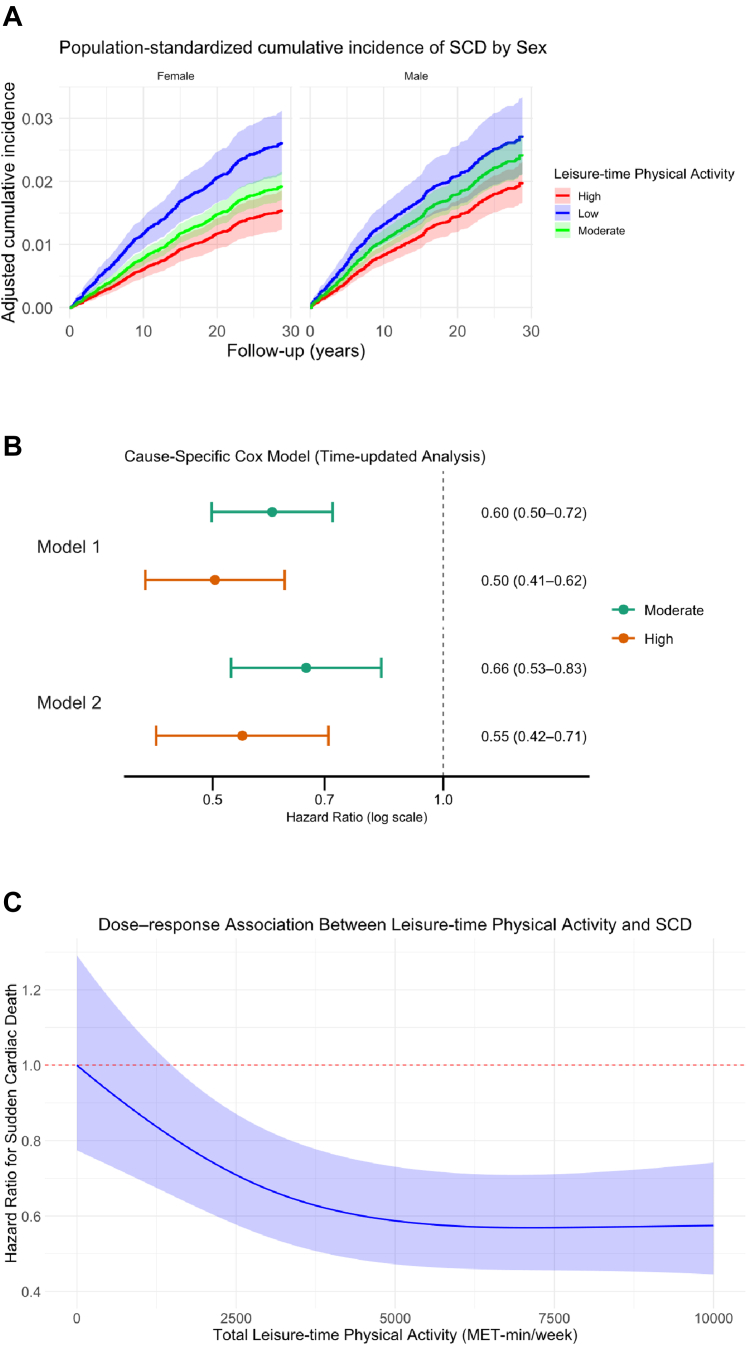
**Population standardized cumulative incidence and hazard ratios of sudden cardiac death by leisure-time physical activity.** (A) Population standardized cumulative incidence of sudden cardiac death with (SCD) 95% confidence intervals according to leisure-time physical activity level. (B) Hazard ratios and 95% confidence intervals for the association between leisure-time physical activity and sudden cardiac death, obtained from time-updated cause-specific Cox proportional hazards models with two model adjustments: Model 1, adjusted for age, sex, alcohol consumption, smoking status and socioeconomic status (income and education). Model 2, additionally adjusted for antihypertensive medication, diabetes, body mass index and cardiovascular disease. (C) Restricted cubic spline curves from a cause-specific Cox proportional hazards model showing the association between total leisure-time physical activity, expressed as MET-minutes per week, and the hazard of SCD. Hazard ratios are presented relative to no leisure-time physical activity (0 MET-min/week). The solid blue line represents the estimated HR, and the shaded area indicates the 95% confidence interval. For interpretation of MET-minute values in terms of leisure-time physical activities, see Supplementary Table S2.

In sex-stratified population-standardized analyses, the cumulative incidence of sudden cardiac death increased progressively with decreasing levels of leisure-time physical activity in both women and men (Fig. 2A). Among women, clearer separation of the cumulative incidence curves across physical activity categories was observed. At 20 years of follow-up, the cumulative incidence of SCD was 1.18% (95% CI 0.94–1.42) in the high, 1.48% (95% CI 1.30–1.66) in the moderate, and 2.07% (95% CI 1.67–2.47) in the low activity group. Among men, a similar graded pattern was present, although the absolute separation between activity categories was less pronounced. At 20 years, cumulative incidence was 1.44% (95% CI 1.20–1.69) in the high, 1.80% (95% CI 1.56–2.04) in the moderate, and 2.09% (95% CI 1.62–2.57) in the low activity group. In CSC models, high activity was associated with a significantly lower risk of SCD compared with low activity (Model 1: HR 0.53, 95% CI 0.43–0.65; p < 0.001). Moderate activity was also associated with a lower risk (Model 1: HR 0.70, 95% CI 0.58–0.84; p < 0.001). Estimates from Model 2 were very similar to those from Model 1 (Supplementary Figure S2). No statistically significant interaction was observed between leisure-time physical activity level and sex (Model 1; p for interaction = 0.37 for moderate activity and 0.52 for high activity), nor between leisure-time physical activity and baseline CVD status (Model 1; p for interaction = 0.56 for moderate activity and 0.62 for high activity). The dose–response curve suggested a non-linear inverse association between leisure-time physical activity and SCD risk, with a steep risk reduction observed from 0 up to approximately 6000 MET-min/week, followed by a plateau at higher activity levels (Fig. 2C). In this cohort, total leisure-time physical activity had a median of 3108 MET-min/week, with an interquartile range of 1596–5276 (Supplementary Figure S3). When categorized, the median (IQR) leisure-time physical activity was 1197 (399–2394) MET-min/week in the low, 2820 (1554–4788) MET-min/week in the moderate, and 4788 (2917–7308) MET-min/week in the high activity group.

### Time-dependent analysis

We next evaluated whether changes in leisure-time physical activity over time influenced the association with SCD. A total of 5007 participants completed the follow-up questionnaire approximately 10 years after baseline. For these individuals, leisure-time physical activity and other time-dependent confounding factors were updated and analyzed as a time-varying covariate. Participants without follow-up data were assumed to have maintained their baseline activity level.

In time-dependent CSC models incorporating updated leisure-time physical activity, the inverse association in moderate activity group was more pronounced compared with baseline-only models (Model 1: HR 0.60, 95% CI 0.50–0.72; p < 0.001) (Fig. 2B). Using the Difference method, the estimated proportion of the association potentially explained by BMI, diabetes, and CVD was 20% for moderate and 12% for high activity, suggesting that these factors account for only a small fraction of the association. Among 4956 participants with complete data at both baseline and follow-up, 1001 increased, 1148 decreased, and 2807 maintained their leisure-time physical activity level. Compared with participants who sustained their leisure-time physical activity, those who increased or decreased activity did not show a statistically significant difference in risk of SCD (HR for Increase 0.95, 95% CI 0.67–1.35; HR for Decrease 0.92, 95% CI 0.68–1.26). Based on Schoenfeld residuals, the proportional hazards assumption was satisfied (Model 1; p = 0.70).

### Population attributable risk

The PAR of SCD associated with low versus high leisure-time physical activity, standardized to the age and sex distribution of the Danish population in 1993 and to the distribution of other covariates in the cohort (smoking, alcohol use, education, and income), was 0.43 (95% CI 0.31–0.55) at 5 years of follow-up, 0.37 (95% CI 0.25–0.49) at 15 years, and 0.33 (95% CI 0.21–0.46) at 25 years.

### Assessment of potential mediators

**Fig. 3:**
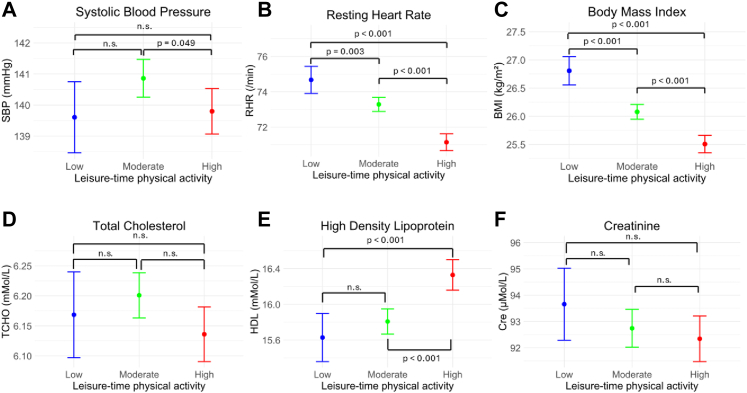
**Associations between leisure-time physical activity and intermediate cardiovascular risk factors.** Adjusted mean values (±95% confidence intervals) of (A) systolic blood pressure, (B) resting heart rate, (C) body mass index, (D) total cholesterol, (E) high-density lipoprotein, and (F) creatinine across categories of leisure-time physical activity are shown. Models were adjusted for age, sex, smoking status, alcohol consumption and socioeconomic status (income and education).

Higher leisure-time physical activity was associated with lower resting heart rate and BMI, with significant differences observed between all activity levels. For systolic blood pressure, only the difference between Moderate and High activity was significant (p = 0.049). Resting heart rate showed a significant inverse trend across activity levels, being lowest in the High group, followed by the Moderate and Low groups. Total cholesterol showed no significant differences, while high density lipoprotein was higher in the High activity group compared with Low or Moderate groups (p < 0.001). Creatinine levels did not differ significantly across activity groups (Fig. 3).

### Sensitivity analyses

We conducted several sensitivity analyses to assess the robustness of our findings. First, a complete-case analysis restricted to participants with complete covariate data (n = 9061) yielded results consistent with the primary analyses, indicating that the use of multiple imputation did not materially influence the estimates (Supplementary Figure S4). Second, when possible SCD cases were excluded, reducing the number of events from 897 to 591, cause-specific Cox models continued to show a significant protective association for high physical activity (Model 1: HR 0.71, 95% CI 0.51–0.98; p = 0.039) (Supplementary Figure S5). Third, excluding SCD events occurring within the first year of follow-up, resulting in 9951 participants available for analysis, produced results that were highly similar to the main findings (Supplementary Figure S6). Fourth, to address potential bias related to missing follow-up information, we repeated the time-updated analyses after restricting the sample to participants who attended the follow-up examination. The hazard ratios for moderate and high leisure-time physical activity were similar to or slightly lower than those observed in the main time-updated analysis (Model 1: Moderate HR 0.51, 95% CI 0.36–0.71; High HR 0.48, 95% CI 0.33–0.70) (Supplementary Figure S7). Together, these analyses support the robustness of the observed associations and suggest that reverse causality or outcome misclassification are unlikely explanations.

## Discussion

This is the first study to evaluate rigorously adjudicated SCD over 28 years of follow-up, while accounting for competing risks and time-varying changes in physical activity. In this large, general population cohort, we observed a clear inverse association between leisure-time physical activity and the risk of SCD. Compared with low activity, high leisure-time physical activity was associated with a substantially lower risk, and moderate activity was also associated with a lower rate of SCD in cause-specific Cox models, even after adjustment for potential confounders. Although sex-stratified cumulative incidence analyses showed a more pronounced separation of curves across leisure-time physical activity categories among women, formal interaction testing in cause-specific Cox models did not provide evidence of effect modification by sex. This suggests that the relative protective association of leisure-time physical activity with SCD is largely consistent in women and men. The greater absolute risk differences observed among women may instead reflect sex-specific baseline characteristics, including differences in age distribution, prevalence of cardiovascular risk factors, and lifestyle profiles, rather than true heterogeneity in the relative effect of physical activity. Importantly, time-dependent analyses using updated information on leisure-time physical activity approximately 10 years after baseline revealed even stronger lower risk associations. While leisure-time physical activity change categories (increase, decrease, sustained) were not significantly associated with SCD risk, these findings suggest that the most recent activity level, rather than long-term changes, may carry the strongest influence on risk. The sensitivity analysis restricted to follow-up responders showed somewhat stronger protective associations for moderate physical activity. This may be attributable to selection bias, as participants who completed the follow-up examination could represent a healthier or more health-conscious subset of the cohort. Such “healthy responder” effects can lead to slight overestimation of the benefits associated with leisure-time physical activity, particularly in groups with moderate levels of activity. However, the overall pattern of risk reduction across activity categories remained consistent, supporting the robustness of our findings. In addition, although underlying cardiovascular disease is present in a large proportion of SCD cases, adjustment for baseline CVD did not materially attenuate the association between leisure-time physical activity and SCD in our study, and no effect modification by CVD status was observed. These findings suggest that the protective association of habitual physical activity with SCD risk extends to individuals both with and without established CVD.

Previous studies have consistently shown that higher physical activity is associated with reduced all-cause and cardiovascular mortality.^30^ We also demonstrated that individuals who were physically active prior to myocardial infarction had a substantially lower risk of fatal myocardial infarction compared with sedentary individuals.^31^ However, most previous studies did not incorporate time-varying assessments of physical activity or explicitly account for competing risks, which may bias risk estimates for cause-specific outcomes, including large population-based cohorts primarily designed to study all-cause or cardiovascular mortality rather than SCD.^30^^,^^31^ Several investigations have also examined the relationship between physical activity and SCD; however, the majority of these studies have focused on exertional SCD occurring during or immediately after vigorous exercise, often among young or competitive athletes,^32^, ^33^, ^34^ rather than on habitual leisure-time physical activity in the general population. In contrast, our study focuses on leisure-time physical activity as a marker of habitual lifestyle, employs systematic manual adjudication of SCD from death certificates, incorporates repeated measurements of physical activity and key covariates, and explicitly addresses competing risks. These methodological features allow a more precise and etiologically relevant assessment of the long-term association between habitual physical activity patterns and SCD risk, thereby extending prior work that has primarily examined all-cause mortality, cardiovascular mortality, or exertion-related SCD.

SCD remains one of the most challenging cardiovascular outcomes to prevent because of its abrupt onset and limited opportunities for acute intervention. In this large, population-based cohort, low leisure-time physical activity was associated with a notable proportion of SCD events. The PAR declined modestly from 43% at 5 years to 33% at 25 years, suggesting a sustained but slightly attenuated preventive potential over time. This gradual decline is likely explained, at least in part, by the increasing influence of competing risks, particularly non-sudden cardiac deaths, among aging participants, rather than a true weakening of the association between leisure-time physical activity and SCD.

Importantly, our dose–response analysis indicated a non-linear relationship between leisure-time physical activity and SCD risk. Risk declined progressively with increasing activity levels up to approximately 6000 MET-min/week, beyond which additional increases in activity were not associated with further risk reduction, suggesting a plateau effect. These findings are broadly consistent with previous studies evaluating cardiovascular disease as the outcome,^35^ which similarly reported that moderate-to-high levels of leisure-time physical activity confer substantial protection, with limited additional benefit at very high activity levels. This implies that substantial protection against SCD may be achieved at moderate-to-high levels of leisure-time physical activity, while extremely high activity does not appear to provide further incremental risk reduction.

Given that leisure-time physical activity is a low-cost, broadly accessible, and modifiable behavior, public health strategies might prioritize interventions and policies that facilitate active lifestyles in the general population. Such initiatives could include urban design promoting active transport and workplace or community programs encouraging regular activity.^36^^,^^37^

Overall, lifelong engagement in leisure-time physical activity appears to be an important component of cardiovascular health promotion and may contribute to a lower population burden of SCD. In future studies, the use of objective measures such as accelerometry or wearable devices could improve precision in quantifying activity levels and tracking changes over time, potentially enhancing risk prediction and intervention design.

In our analyses, further adjustment for potential mediators including BMI, cardiovascular disease, and diabetes only modestly attenuated the association between physical activity and SCD, suggesting that these cardiometabolic factors do not fully account for the observed relationship. We also observed that higher leisure-time physical activity was associated with lower resting heart rate, lower BMI, and higher HDL cholesterol (Fig. 3). These factors may partly reflect improved autonomic regulation, cardiorespiratory fitness, and lipid metabolism among more physically active individuals. Accordingly, regular leisure-time physical activity may reduce the risk of sudden cardiac death through multiple interrelated physiological pathways.

One important mechanism involves autonomic regulation. Lower resting heart rate among physically active individuals may reflect enhanced vagal modulation and reduced sympathetic drive, indicating a more favorable autonomic balance. Experimental and translational studies have shown that regular physical activity increases resting vagal tone, which is known to confer protection against malignant ventricular arrhythmias.^38^

In addition to autonomic effects, physical activity is associated with more favorable metabolic and vascular profiles. Lower body mass index and higher levels of HDL cholesterol among physically active individuals may indicate improved glucose homeostasis, blood pressure regulation, and reduced systemic inflammation, alongside enhanced lipid transport and endothelial function. However, emerging evidence suggests that the cardiovascular protective effects of HDL cholesterol may be attenuated in the presence of obesity, highlighting the importance of body composition in modulating lipid-related risk.^39^

Beyond traditional cardiometabolic pathways, accumulating evidence indicates that habitual physical activity improves cardiorespiratory fitness and exercise tolerance, which may increase the workload threshold at which myocardial ischemia and malignant ventricular arrhythmias are triggered.^5^ Higher fitness levels are associated with greater ischemic tolerance, such that a higher myocardial oxygen demand is required to provoke ischemia or arrhythmic events, thereby reducing the likelihood of SCD during daily activities. Experimental studies in animal models provide mechanistic support for this concept. In post myocardial infarction canine models, daily exercise training has been shown to eliminate ventricular fibrillation during acute ischemia in previously susceptible animals, accompanied by improvements in baroreflex sensitivity and vagal modulation, suggesting an increased arrhythmic threshold.^40^ In addition, both short- and long-term aerobic training enhance myocardial tolerance to ischemia–reperfusion injury, reduce infarct size, and improve contractile function through multiple pathways, including upregulation of heat shock proteins, nitric oxide–protein kinase C–K_ATP channel signaling, activation of survival kinases, antioxidant defenses, and favorable mitochondrial and metabolic adaptations.^41^^,^^42^

Taken together, these results suggest that although part of the protective association of physical activity with SCD may be mediated through improvements in autonomic function, body composition, and lipid profile, the remaining association may involve additional mechanisms beyond traditional cardiometabolic pathways. These may include fitness-related adaptations, such as higher ischemic and arrhythmic thresholds, as suggested by experimental and clinical studies.

This study has several limitations. First, leisure-time physical activity was self-reported, which may introduce recall or reporting bias; for example, participants might overestimate their leisure-time activity, potentially leading to under- or overestimation of the true association with SCD. Second, the observational nature of the study precludes causal inferences, and residual confounding remains possible despite adjustment for multiple covariates. While age, sex, and socioeconomic status were accounted for, other unmeasured confounders such as genetic predisposition, dietary factors, psychosocial stress, medication use, environmental or sleep characteristics could influence both physical activity and SCD risk. Only two time points of physical activity were assessed, which may not fully capture long term changes, intensity, or patterns of activity over the life course. Work related physical activity and specific types of exercise, such as cardiorespiratory or strength training, were not separately evaluated, although they may have differential effects on cardiovascular risk. Moreover, the age distribution of the cohort is skewed toward older adults, with a relative underrepresentation of younger individuals. Consequently, SCD events driven by causes more prevalent in younger populations, such as primary arrhythmias, may be underrepresented, whereas events related to ischemic heart disease are more frequent. This limits the generalizability of the findings to younger populations. Finally, the study was conducted in an urban area of the Danish capital region, and findings may not be generalizable to rural populations or other countries with different lifestyle, socioeconomic, or healthcare contexts.

## Contributors

T.S., R.J and J.T.-H. contributed to the acquisition of the data. S.I., T.S., P.E.W. and J.T.-H. conceptualized the study. S.I., T.S. and P.E.W. performed the formal analysis. S.I., T.S., E.C., P.E.W. and J.M. drafted the manuscript. E.I.B.S. and J.T.-H. supervised the work. S.I. and T.S. accessed and verified the data. All authors critically revised the manuscript, assisted in the interpretation of data, and gave final approval for submission.

## Data sharing statement

Data are not publicly available but may be obtained after a formal request to and acceptance by the Copenhagen City Heart Study and the Danish Data Protection Agency.

## Declaration of interests

J.T.-H. has been consultant for Cytokinetics, Solid BioScience and Boston Scientific. J.T.-H. (Payment or honoraria for lectures, presentations, speakers bureaus, manuscript writing or educational events) for Johnson and Johnson. J.T.-H. (Payment or honoraria for lectures, presentations, speakers bureaus, manuscript writing or educational events) for Microport. J.T.-H. (Payment or honoraria for lectures, presentations, speakers bureaus, manuscript writing or educational events) for Abbott.

## Appendix A Supplementary data

Supplementary Tables and Figures

Japanese summary
