# Supplementary material for: Leisure-time physical activity and risk of sudden cardiac death: a 28-year follow-up from the Copenhagen City Heart Study
Source: eClinicalMedicine. 2026 Mar 10;93:103825. doi: 10.1016/j.eclinm.2026.103825 (PMC12994074; doi:10.1016/j.eclinm.2026.103825)
Supplement: Supplementary Tables and Figures [file mmc1.docx]

**Supplementary Table 1: Missing Data Summary**

| Variable | Missing (n, %) |
| --- | --- |
| Physical activity | 145 (1.4%) |
| smoking | 62 (0.6%) |
| Alcohol | 135 (1.3%) |
| Income | 342 (3.4%) |
| Education | 351 (3.5%) |
| Antihypertensive medication | 122(1.2%) |
| Diabetes | 72(0.7%) |
| BMI | 415(4.1%) |
| Prior myocardial infarction | 53(0.5%) |

**Supplementary Table 2:** **MET values assigned to each leisure-time physical activity**

| **Activity** | **MET** |
| --- | --- |
| Walking (light) | 3.0 |
| Walking (moderate) | 3.8 |
| Walking (brisk) | 4.8 |
| Walking (fast) | 5.5 |
| Cycling (leisure) | 6.8 |
| Cycling (moderate) | 8.0 |
| Cycling (vigorous) | 10.0 |
| Cycling (fast) | 12.0 |
| Running | 13.3 |
| Gymnastics | 3.8 |
| Swimming | 4.8 |
| Tennis | 6.8 |
| Badminton | 5.5 |
| Soccer | 7.0 |
| Handball | 12.0 |
| Health-oriented exercise | 2.8 |
| Weightlifting | 6.0 |

**Supplemental Figure 1**


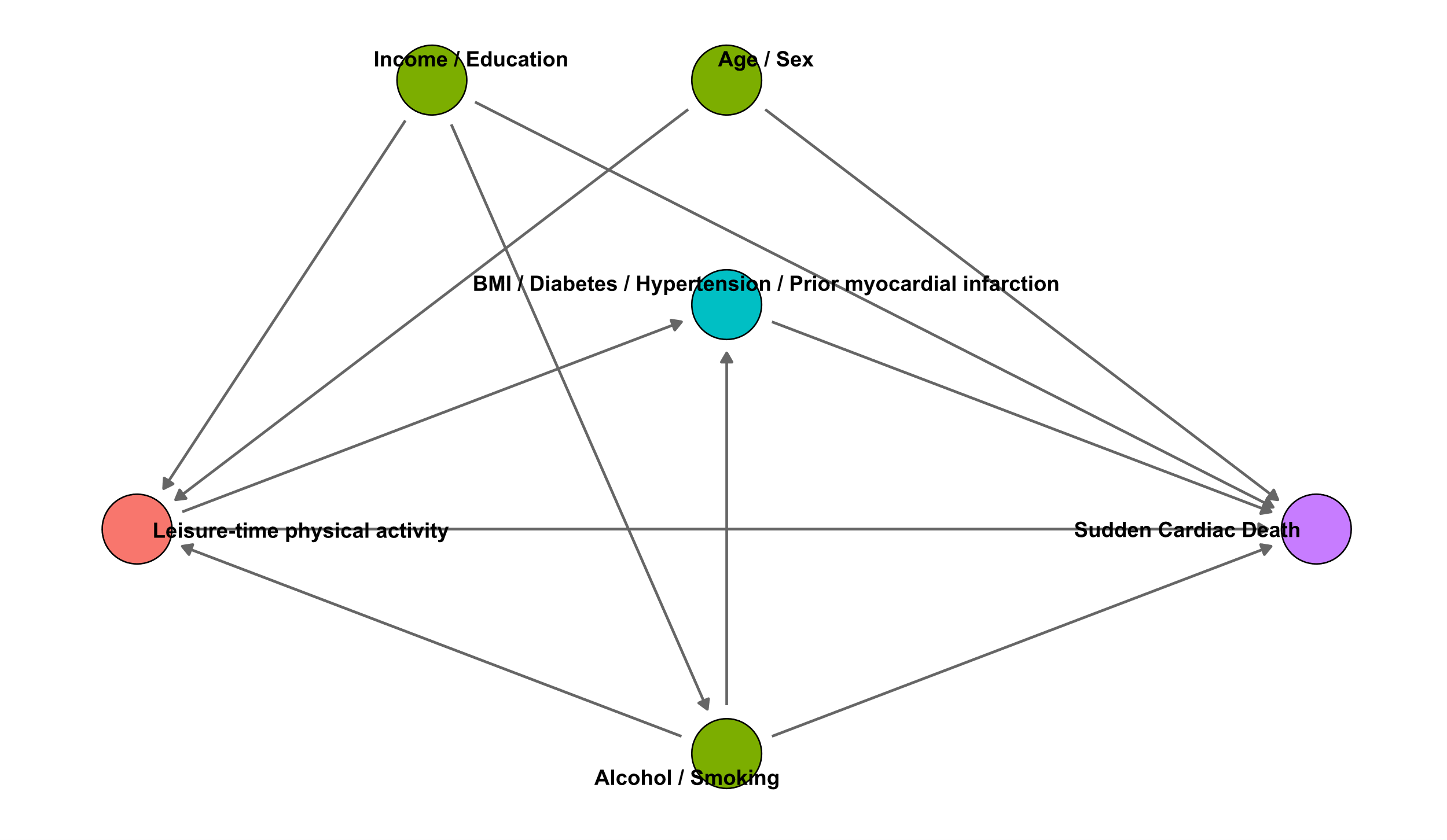
 Directed acyclic graph (DAG) of the association between leisure-time physical activity and sudden cardiac death. Red nodes indicate the exposure (leisure-time physical activity), purple nodes indicate the outcome (sudden cardiac death), green nodes indicate confounders, and sky-blue nodes indicate potential mediators.

**Supplemental Figure 2**

Hazard ratios with 95% confidence intervals for the association between baseline leisure-time physical activity and sudden cardiac death, estimated from cause-specific Cox models using baseline activity only. Estimates were obtained from cause-specific Cox proportional hazards models with two model adjustments: Model 1, adjusted for age, sex, alcohol consumption, smoking status and socioeconomic status (income and education). Model 2, additionally adjusted for cardiovascular disease, diabetes and body mass index.
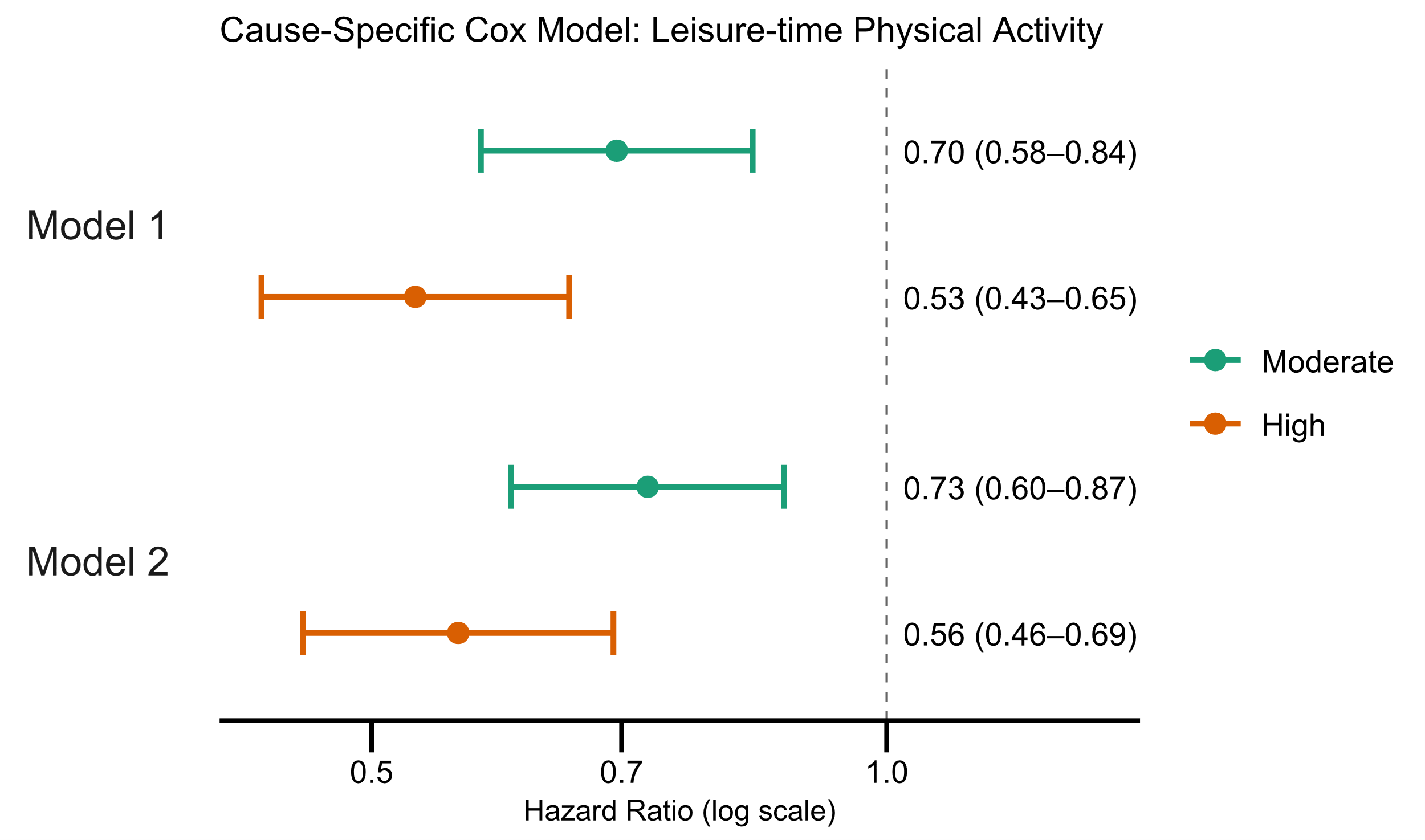


**Supplemental Figure 3**

Histogram showing the distribution of total leisure-time physical activity in MET-minutes per week among study participants. The x-axis represents total LTPA per week, and the y-axis represents the number of participants.

**
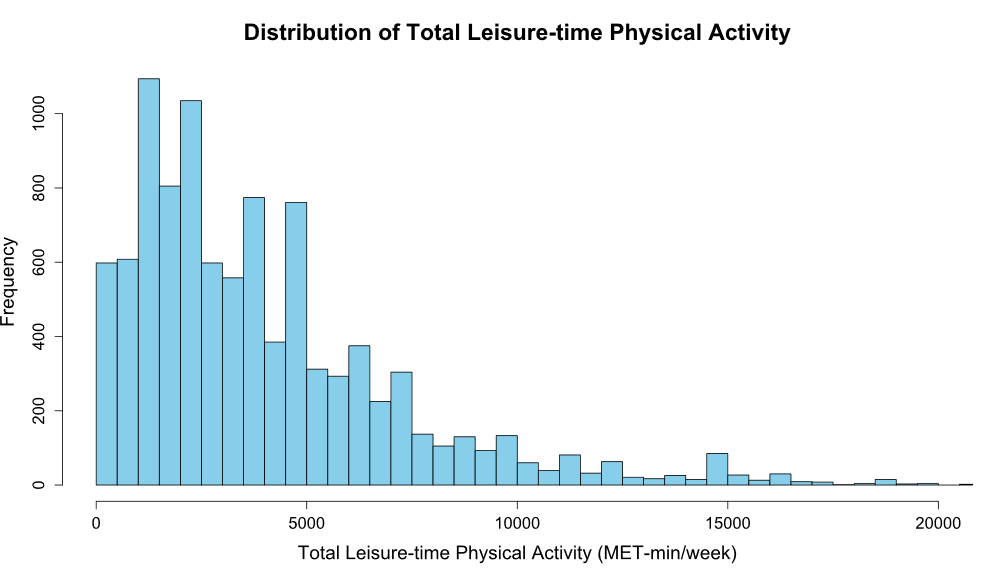
**

**Supplemental Figure 4**

Hazard ratios and 95% confidence intervals for the association between leisure-time physical activity and sudden cardiac death for complete cases (n = 9061). Estimates were obtained from cause-specific Cox proportional hazards models with two model adjustments: Model 1, adjusted for age, sex, alcohol consumption, smoking status and socioeconomic status (income and education). Model 2, additionally adjusted for cardiovascular disease, diabetes and body mass index.


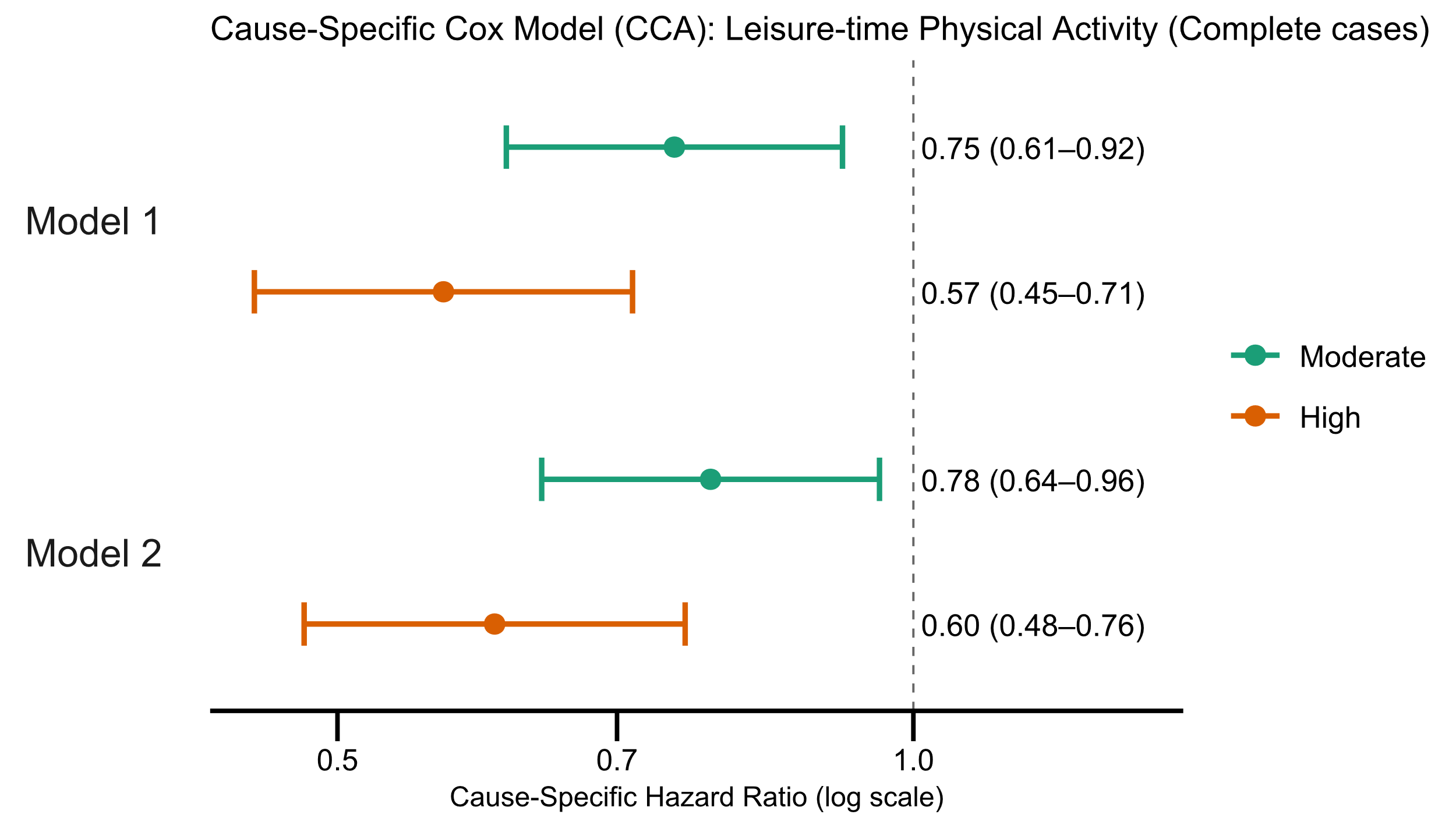
.

**Supplemental Figure 5**

Hazard ratios and 95% confidence intervals for the association between leisure-time physical activity and sudden cardiac death for limited only define and probable sudden cardiac death cases. Estimates were obtained from cause-specific Cox proportional hazards models with two model adjustments: Model 1, adjusted for age, sex, alcohol consumption, smoking status and socioeconomic status (income and education). Model 2, additionally adjusted for cardiovascular disease, diabetes and body mass index.


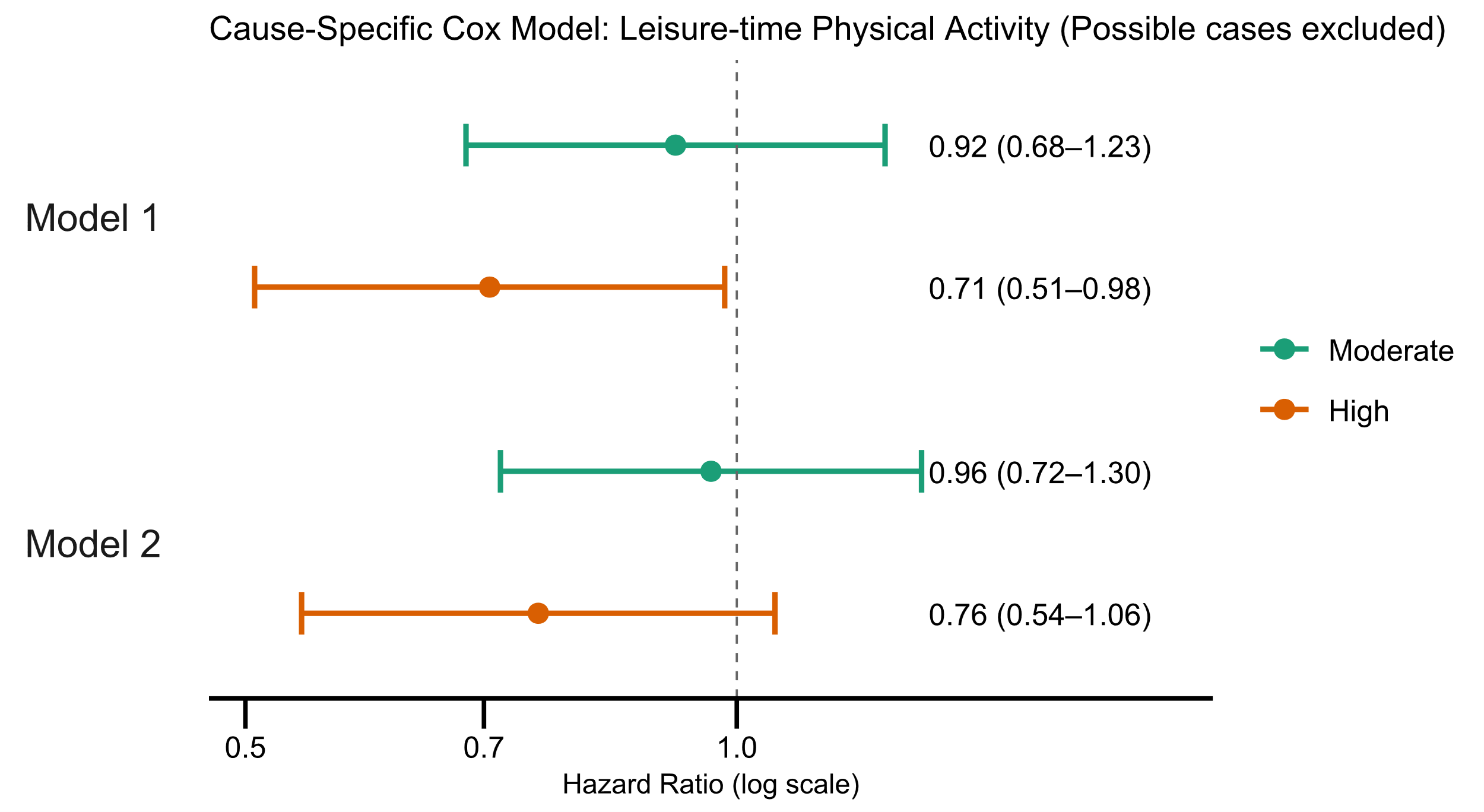


**Supplemental Figure 6**

Hazard ratios and 95% confidence intervals for the association between leisure-time physical activity and sudden cardiac death, excluded sudden cardiac death events occurring within the first year. Estimates were obtained from cause-specific Cox proportional hazards models with two model adjustments: Model 1, adjusted for age, sex, alcohol consumption, smoking status and socioeconomic status (income and education). Model 2, additionally adjusted for cardiovascular disease, diabetes and body mass index.


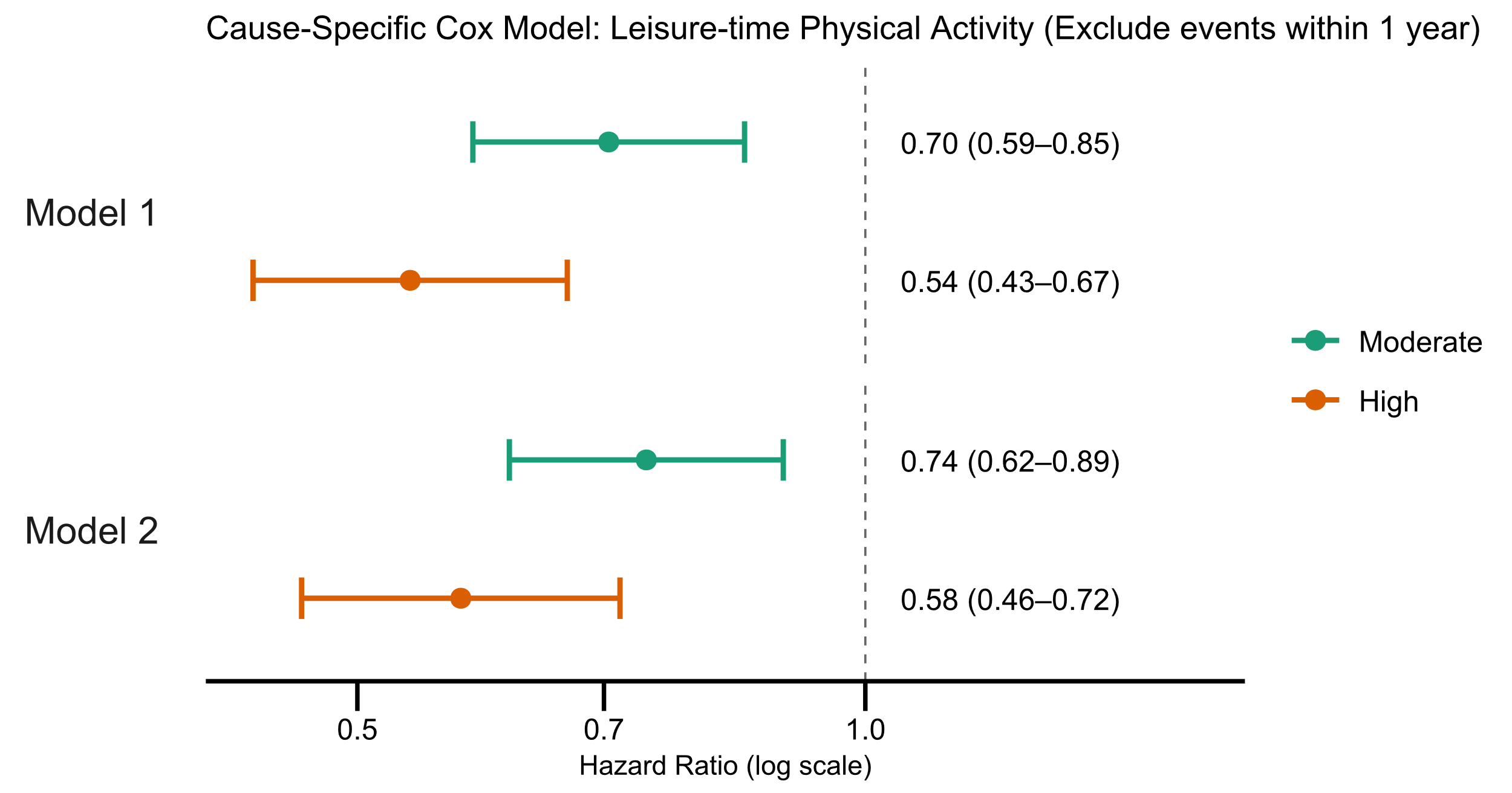


**Supplemental Figure 7**

Hazard ratios with 95% confidence intervals for the association between baseline leisure-time physical activity and sudden cardiac death among participants who responded to the follow-up questionnaire. Estimates were obtained from cause-specific Cox proportional hazards models with two model adjustments: Model 1, adjusted for age, sex, alcohol consumption, smoking status and socioeconomic status (income and education). Model 2, additionally adjusted for cardiovascular disease, diabetes and body mass index.

**
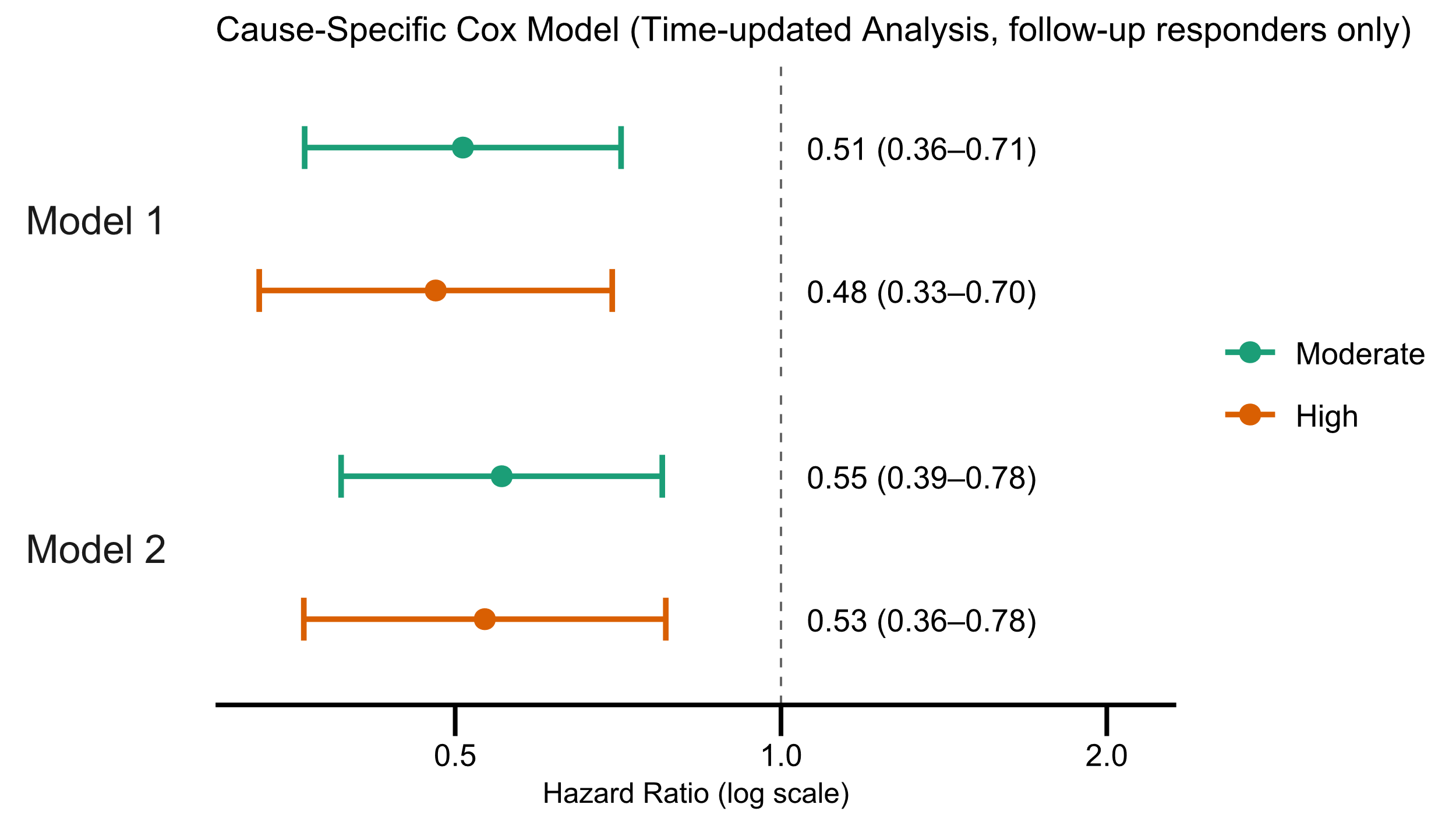
**

**Supplementary Method 1**

**Definition of SCD**

- **Definite sudden death (SD):** Sudden, natural, and unexpected death with a clearly established time frame: in witnessed cases, an acute change in cardiovascular status leading to death within 1 hour; in unwitnessed cases, last seen alive and functioning normally within 24 hours before being found dead.
- **Probable SD:** No fully documented time frame from symptom onset to death, but available evidence strongly suggested a sudden death, in the absence of severe or chronic illnesses expected to cause a non-sudden death. Cases with advanced decomposition were excluded as non-SD.
- **Definite SCD:** Definite SD with either an autopsy-confirmed cardiac or arrhythmic cause of death or documented ventricular arrhythmia immediately preceding death.
- **Probable SCD:** Definite SD deemed likely of cardiac origin.
- **Possible SCD:** Probable SD with less certain but suspected cardiac origin.

**Supplementary Method 2**

**The questionnaires to check leisure-time physical activity levels in Copenhagen City Heart Study.**

Questions: Over the past year, which of the following best describes your leisure-time physical activity (including commuting)?

Answer:

1. Almost no exercise or <2h/week light activity (reading, TV, movies)
2. 2–4h/week light activity (walking, light cycling, light gardening, light gymnastics)
3. >4h/week light activity or 2–4h/week vigorous activity (brisk walking, fast cycling, strenuous gymnastics)
4. >4h/week vigorous activity, regular hard training or competitions several times a week

Responses were categorized into three levels of leisure-time physical activity: “Low” (answer 1), “Moderate” (answer 2), and “High” (answers 3 and 4).
