# Supplementary material for: Leisure-time physical activity and risk of sudden cardiac death: a 28-year follow-up from the Copenhagen City Heart Study
Source: eClinicalMedicine. 2026 Mar 10;93:103825. doi: 10.1016/j.eclinm.2026.103825 (PMC12994074; doi:10.1016/j.eclinm.2026.103825)
Supplement: Japanese summary [file mmc2.docx]

*The following translations in Japanese were submitted by the authors and we reproduce them as supplied. They have not been peer reviewed. Our editorial processes have only been applied to the original abstract in English, which should serve as reference for this manuscript.*

余暇時身体活動と心臓性突然死の長期リスク：コペンハーゲン心臓研究

**背景**

心臓性突然死（SCD）は依然として重要な公衆衛生上の課題であるが、余暇時身体活動とSCDリスクとの長期的関連は十分に明らかになっていない。本研究では、余暇時身体活動と長期にわたるSCD発症との関連を、競合リスクおよび経時的な身体活動の変化を考慮して検討した。

**方法**

1991～1994年に調査されたコペンハーゲン心臓研究の参加者10,100人を対象とし、2021年12月31日まで追跡した。CPRナンバーによる個人単位での紐づけを用いて死亡調査票を全例精査し、死因をSCDと非SCDに分類した。余暇時身体活動はベースラインおよび10年後追跡時に自己申告で評価し、時間更新変数として解析に組み入れた。SCDとの関連は、年齢、性別、喫煙、飲酒、社会経済的要因で調整した原因特異的Cox比例ハザードモデルにより推定した。累積発症率はデンマーク人口に標準化し、低身体活動に起因する人口寄与危険割合を算出した。

**結果**

追跡期間中央値28.6年の間に、10,100人（平均年齢60.8歳、女性56%）中897例のSCDが発生した。20年間の標準化累積発症率は、身体活動レベルが低いほど高値を示した。時間更新解析では、中等度および高い余暇時身体活動は低活動と比較してSCDリスクの低下と関連していた（中等度：ハザード比0.60、95%信頼区間0.50–0.72；高度：0.50、95%信頼区間0.41–0.62）。人口寄与危険割合の算出により、25年時点でSCDの33%（95%信頼区間21–46%）が低身体活動に起因すると推定された。

**公衆衛生上の示唆**

本観察研究において、余暇時身体活動量が高いことはSCDの長期リスク低下と関連していた。因果関係を直接示すものではないが、本研究結果はSCD予防戦略における余暇時身体活動促進の重要性を浮き彫りにしている。
